# Assessing the Quality of World Health Organisation Guidelines during Health Emergencies: A Domain-Based Analysis

**DOI:** 10.1007/s44197-025-00461-3

**Published:** 2025-10-10

**Authors:** Bernard Ayine, Cornelius Fuumaale Suom-Kogle

**Affiliations:** 1 The Hong Kong Polytechnic University, Kowloon, Hong Kong; 2 Michigan Technological University, Houghton, USA

**Keywords:** Disease outbreak news, Emergencies, Guidelines, Health risks, World health organisation

## Abstract

**Background:**

Effective response during global health emergencies hinges on the quality of guidelines provided by authoritative organisations like the World Health Organisation (WHO). This study assessed the quality of WHO emergency guidelines disseminated through the Disease Outbreak News (DONs) platform between 2023 and 2024 to identify strengths and weaknesses across established quality domains.

**Methods:**

A total of 115 WHO guidelines issued within DONs were analysed using the AGREE II framework, which evaluates six domains: Scope and Purpose; Stakeholder Involvement; Rigour of Development; Clarity of Presentation; Applicability, and Editorial Independence. Descriptive statistics and one-way repeated measures ANOVA were conducted to determine significant differences among domain scores.

**Results:**

The analysis revealed statistically significant differences across domains, *F*(2.40, 552.34) = 739.09, *p* < .001, *ηp²* = 0.866. The highest mean scores were recorded for Scope and Purpose (*M* = 6.46) and Clarity of Presentation (*M* = 6.27), indicating strengths in goal articulation and user accessibility. Conversely, Editorial Independence (*M* = 2.74) and Rigour of Development (*M* = 3.26) scored the lowest, pointing to persistent gaps in transparency and methodological robustness.

**Conclusions:**

While WHO guidelines during emergencies perform well in clarity and scope, critical weaknesses remain in transparency, stakeholder engagement, and methodological rigour. These findings indicate the need for more balanced and inclusive guideline development processes to enhance trust and utility during public health emergencies.

## Introduction

The rising frequency and complexity of regional and global health emergencies, such as Mpox, Nipah virus infection, and Influenza A (H1N1) variant virus, have pointed to the urgent need for more effective communication and coordination between international agencies and national or regional bodies to facilitate timely containment and reduce public health risks [1–4]. The outbreak of an infectious disease imposes demands on governments and health institutions to make decisions aimed at protecting public health and minimising harm [5]. These decisions affect both local and global health systems. In such high-pressure environments, often marked by scientific uncertainty and evolving threats, reliable, evidence-based guidelines become indispensable tools for decision-making [6, 7]. One of the central actors in global health governance during such crises is the World Health Organisation (WHO), which provides authoritative guidance to its 192 member states (excluding the United States, which exited the WHO in 2025) through the publication of public health and clinical guidelines to facilitate surveillance, clinical care, vaccination strategies, and public communication [1, 3, 6].

In line with its commitment to global health security, the WHO operates the Disease Outbreak News (DONs) platform, which is established under Article 11.4 of the International Health Regulations (2005). This platform provides real-time updates on emerging health threats and typically includes six sections, the final of which presents guidance for addressing the emergency. DONs are intended for a wide audience, including the general public, policymakers, healthcare professionals, and national health authorities, and are vital in guiding response efforts during times of heightened uncertainty [5, 7]. The effectiveness of these guidelines is especially critical when outbreaks unfold rapidly and reliable information is scarce [8]. During the Ebola outbreak, for example, local and global actors struggled to deliver timely and consistent interventions. The delayed release of a formal response plan by the WHO, nearly five months after the first cross-border transmission, and poor communication contributed to intensified public anxiety and accelerated disease spread [9, 10].

Despite the reputation of the WHO as a leader in global health, the organisation has not been without criticism. Over a decade ago, concerns were raised regarding the quality and trustworthiness of some of its published guidelines, particularly those developed under emergency conditions [11]. In response, the WHO established the Guideline Review Committee (GRC) to ensure that all guidelines are grounded in evidence, developed through transparent and systematic processes, and adhere to the highest international standards. Garritty et al. [6] have detailed the steps involved in guideline formulation by highlighting key principles such as transparency, methodological rigour, and bias reduction. These principles formalised in the WHO *Handbook for Guideline Development* [12] are intended to ensure that WHO guidelines are both credible and useful to end-users. However, empirical evaluations of the extent to which these principles are reflected in actual emergency guidelines remain limited.

This study builds on existing research on guideline development [13] by evaluating how well WHO guidelines, particularly those embedded within DONs, align with these established standards. In doing so, it responds to calls by scholars such as Norris et al. [11, p. 2]] who argue that “WHO guidelines produced in the context of a public health emergency can be improved upon, helping to assure the trustworthiness and utility of WHO information products in future emergencies.” Similar sentiments have been expressed by Saxena et al. [14], who intimate the need for ongoing revisions to improve the responsiveness and relevance of WHO guidelines in rapidly evolving situations. In a study conducted by Taylor et al. [15] of 29 WHO guidelines on maternal and newborn health (MANH) and infectious diseases published from 2020 to 2022, the authors argued that WHO frequently employed the use of qualitative evidence in the formulation of guidelines. These guidelines were descriptive in nature and “No guideline provided transparent reporting of how qualitative research was interpreted, weighed and used alongside other evidence when informing decisions, and only one guideline reported the inclusion of qualitative methods experts on the panel” [15, p. 1]]. These findings highlight the current lapses in WHO guidelines.

## Methods

**Fig. 1:**
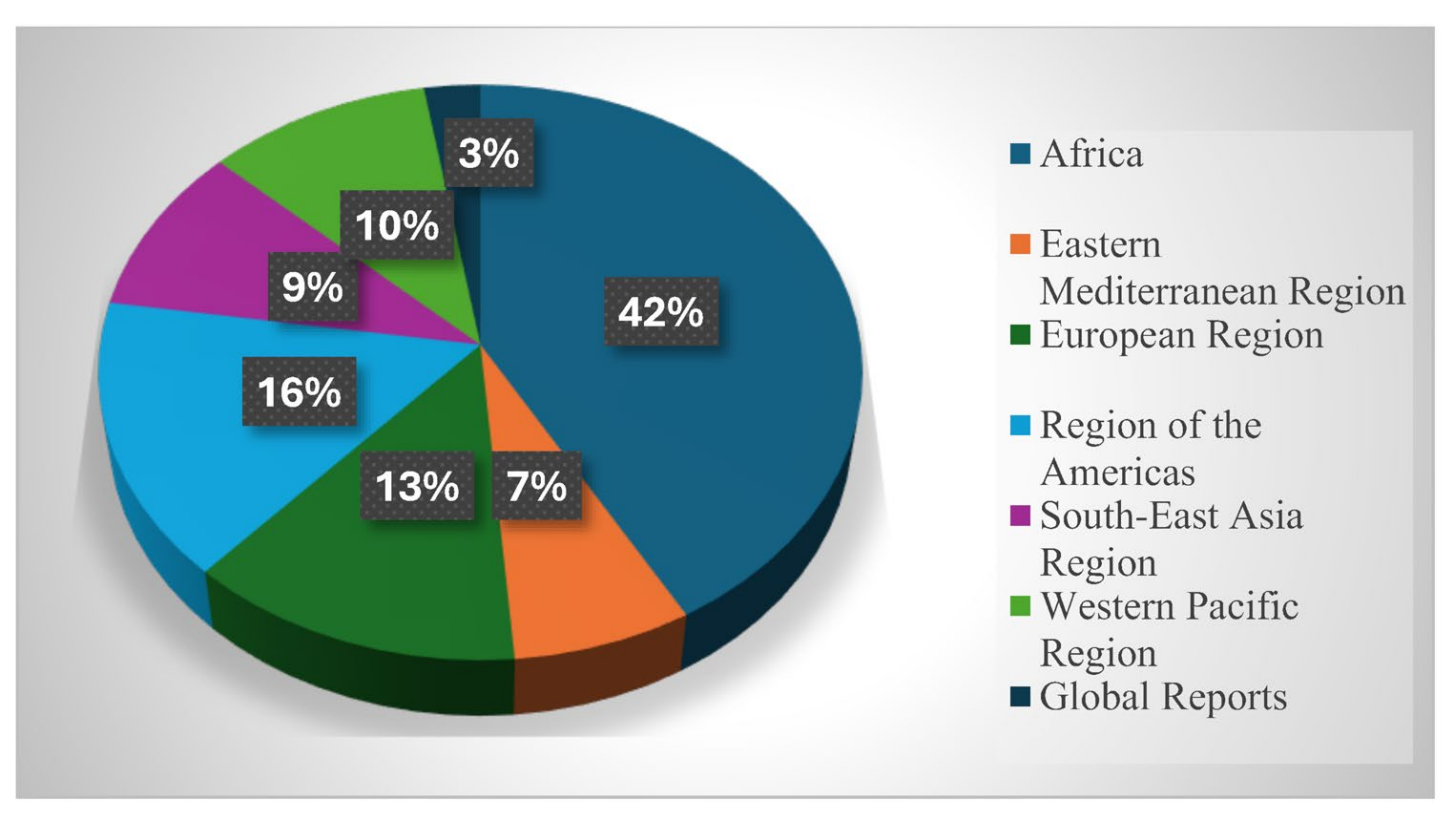
Regional distribution of World Health Organisation Disease Outbreak News (*N* = 115) published from 2023 to 2024

DONs are the “the most frequently used written outlet (and the only official public record of outbreak history curated by the WHO)” [1, p. 2]] that serve as central communication tools and guidance documents in global health governance [16]. For the purpose of this study, the “WHO Advice” section within the DONs were analysed, as it typically provides a set of guidelines directed at the public, health authorities, and other stakeholders on how to manage the specific outbreak. This section was identified as the most appropriate focus for evaluating the quality and utility of WHO’s emergency guidance. A total of 115 Disease Outbreak News reports were collected from the official WHO website under the Emergencies section (https://www.who.int/emergencies/disease-outbreak-news). The sample includes all DONs published by the WHO between 2023 and 2024, covering both global and regional health emergencies. Figure 1 below presents the regional distribution of the DONs included in the analysis.

As shown in Fig. 1, the African Region recorded the highest number of Disease Outbreak News (DONs), accounting for 42% of all reports issued by the WHO between 2023 and 2024. In contrast, the Eastern Mediterranean Region had the lowest number of reports, with only eight cases (7%) documented during the same period. There were three (3) cases in the data where DONs issued between 2023 and 2024 were directed to the global community rather than to any specific region.

The data for this study were compiled into a single document and independently coded by two coders using the *Appraisal of Guidelines for Research and Evaluation* (AGREE) instrument. Specifically, the revised AGREE II tool developed by Brouwers et al. [17] was employed. This instrument is an updated version of the original AGREE tool introduced in 2003 by a consortium of researchers and guideline developers. The original version comprised 23 items aimed at “assessing the process of guideline development and the reporting of this process in the guideline” [17, p. 839]]. AGREE II retains the foundational structure of its predecessor while incorporating key refinements to improve the clarity and comprehensiveness of individual items. Each item is rated on a seven-point Likert scale, allowing for detailed assessment. The instrument has been widely validated and is considered a reliable tool for evaluating clinical practice guidelines (CPGs) [18]. It has seen extensive use in various health domains, including cancer control, and has demonstrated acceptable psychometric properties [17, 19, 20].

AGREE II evaluates guidelines across six core domains: (1) Scope and Purpose, (2) Stakeholder Involvement, (3) Rigour of Development, (4) Clarity of Presentation, (5) Applicability, and (6) Editorial Independence. These domains are essential for understanding, applying, and critically evaluating the overall quality of health-related guidelines, particularly within the context of disease outbreaks. The individual items of each of domains in the instrument are presented in Table 1 below. The full AGREE II instrument and accompanying documentation are publicly accessible at www.agreetrust.org.

**Table 1:** Domains and items of the AGREE II instrument (Updated in December 2017)

Domain	Item
Scope and Purpose	1. The overall objective (s) of the guideline is (are) specifically described.
	2. The health questions (s) covered by the guideline is (are) specifically described
	3. The population (patients, public etc.) to whom the guideline is meant to apply is specifically described
Stakeholder Engagement	4. The guideline development group includes individuals from all relevant professional groups.
	5. The views and preferences of the target population (patients, public, etc.) have been sought.
	6. The target users of the guideline are clearly defined.
Rigour of Development	7. Systematic methods were used to search for evidence.
	8. The criteria for selecting the evidence are clearly described.
	9. The strengths and limitations of the body of evidence are clearly described.
	10. The methods for formulating the recommendations are clearly described.
	11. The health benefits, side effects, and risks have been considered in formulating the recommendations.
	12. There is an explicit link between the recommendations and the supporting evidence.
	13. The guideline has been externally reviewed by experts prior to its publication.
	14. A procedure for updating the guideline is provided.
Clarity of Presentation	15. The recommendations are specific and unambiguous.
	16. The different options for management of the condition or health issue are clearly presented.
	17. Key recommendations are easily identifiable.
Applicability	18. The guideline describes facilitators and barriers to its application.
	19. The guideline provides advice and/or tools on how the recommendations can be put into practice.
	20. The potential resource implications of applying the recommendations have been considered.
	21. The guideline presents monitoring and/or auditing criteria.
Editorial Independence	22. The views of the funding body have not influenced the content of the guideline.
	23. Competing interests of guideline development group members have been recorded and addressed.

The first round of coding involved a sample of 30 DONs. According to O’Connor and Joffe (2020), 10 to 25% of the total data units is usually acceptable for this kind of coding. Two coders independently conducted the initial coding of these guidelines. Inter-coder reliability was assessed using percentage of agreement, which indicated an overall agreement rate of 71.74%. Inter-coder reliability was substantial [21]. Any discrepancies in the coding process were resolved through discussion. Once consensus was reached, the first author proceeded to code the remaining data.

Given that the dataset comprised a single independent variable with one level, a one-way repeated measures ANOVA was deemed appropriate for comparing the mean scores across the different domains of the guidelines [22]. Prior to conducting the analysis, the data were assessed for normality using Q–Q plots and the Shapiro–Wilk test. The results indicated that the assumption of normality was satisfied. The assumption of sphericity was tested using Mauchly’s Test of Sphericity.

In this analysis, the WHO guidelines functioned as the independent variable, while the six (6) domains of the AGREE II instrument were treated as the dependent variables. The significance level was set at α = 0.05 (two-tailed) for all statistical tests. All analyses were performed using IBM SPSS Statistics (version 20).

## Results

Table 2 below presents the descriptive statistics of the six domains (6) of the 115 WHO guidelines analysed in this study. As shown in the table, the highest mean score was observed in the domain Scope and development (*M* = 6.46, *SD* = 0.45), followed by Clarity of presentation (*M* = 6.27, *SD* = 0.68). The domain with the least score was Editorial independence (*M* = 2.74, *SD* = 1.02).

**Table 2:** Descriptive statistics of guideline domains

Domain	M	SD
Scope and purpose	6.46	0.45
Stakeholder involvement	4.50	0.69
Rigour of development	3.26	0.75
Clarity of presentation	6.27	0.68
Applicability	4.48	0.70
Editorial independence	2.74	1.02

A one-way repeated measures ANOVA was conducted to compare the mean scores of WHO guidelines across all six domains. Mauchly’s test of sphericity indicated a violation of the sphericity assumption, *χ²(*14) = 257.66, *p* < .001. Consequently, the degrees of freedom were adjusted using Greenhouse-Geisser estimates (ε = 0.481). The analysis revealed a statistically significant difference in mean scores across the six domains, *F*(2.40, 552.34) = 739.09, *p* < .001, *ηp*² = 0.866, indicating a large effect size [23].

**Table 3:** Result of post hoc comparisons for guideline domains

(I) Domain	(J) Domain	Mean Difference (I-J)	Std. Error	Sig.^b^	95% Confidence Interval for Difference^b^	
					Lower	Upper
Scope and purpose	Stakeholder involvement	1.962^*^	0.053	0.000	1.803	2.122
	Rigour of development	3.208^*^	0.059	0.000	3.032	3.384
	Clarity of presentation	0.191^*^	0.052	0.006	0.034	0.348
	Applicability	1.983^*^	0.050	0.000	1.834	2.132
	Editorial independence	3.720^*^	0.099	0.000	3.422	4.018
Stakeholder Involvement	Scope and purpose	-1.962^*^	0.053	0.000	-2.122	-1.803
	Rigour of development	1.246^*^	0.049	0.000	1.098	1.394
	Clarity of presentation	-1.771^*^	0.067	0.000	-1.972	-1.570
	Applicability	0.021	0.042	1.000	− 0.105	0.147
	Editorial independence	1.758^*^	0.112	0.000	1.424	2.092
Rigour of development	Scope and purpose	-3.208^*^	0.059	0.000	-3.384	-3.032
	Stakeholder involvement	-1.246^*^	0.049	0.000	-1.394	-1.098
	Clarity of presentation	-3.017^*^	0.075	0.000	-3.241	-2.793
	Applicability	-1.225^*^	0.051	0.000	-1.378	-1.072
	Editorial independence	0.512^*^	0.117	0.000	0.160	0.864
Clarity of presentation	Scope and purpose	− 0.191^*^	0.052	0.006	− 0.348	− 0.034
	Stakeholder involvement	1.771^*^	0.067	0.000	1.570	1.972
	Rigour of development	3.017^*^	0.075	0.000	2.793	3.241
	Applicability	1.792^*^	0.061	0.000	1.609	1.975
	Editorial independence	3.529^*^	0.119	0.000	3.173	3.885
Applicability	Scope and purpose	-1.983^*^	0.050	0.000	-2.132	-1.834
	Stakeholder involvement	− 0.021	0.042	1.000	− 0.147	0.105
	Rigour of development	1.225^*^	0.051	0.000	1.072	1.378
	Clarity of presentation	-1.792^*^	0.061	0.000	-1.975	-1.609
	Editorial independence	1.737^*^	0.107	0.000	1.416	2.058
Editorial independence	Scope and purpose	-3.720^*^	0.099	0.000	-4.018	-3.422
	Stakeholder involvement	-1.758^*^	0.112	0.000	-2.092	-1.424
	Rigour of development	− 0.512^*^	0.117	0.000	− 0.864	− 0.160
	Clarity of presentation	-3.529^*^	0.119	0.000	-3.885	-3.173
	Applicability	-1.737^*^	0.107	0.000	-2.058	-1.416

*. The mean difference is significant at the 0.05 level

A post hoc comparison using the Bonferroni correction was conducted to examine the significance of mean differences across domains. As presented in Table 3 above, the results revealed several statistically significant differences across the domains. Scope and development scored significantly higher than Stakeholder involvement (*MD* = 1.96, *SE* = 0.05, *p* < .001, 95% *CI* [1.80, 2.12]), Rigour of development (*MD* = 3.21, *SE* = 0.06, *p* < .001, 95% *CI* [3.03, 3.38]), Clarity of presentation (*MD* = 0.19, *SE* = 0.05, *p* = .006, 95% CI [0.03, 0.35]), Applicability (*MD* = 1.98, *SE* = 0.05, *p* < .001, 95% *CI* [1.83, 2.13]), and Editorial independence (*MD* = 3.72, *SE* = 0.10, *p* < .001, 95% *CI* [3.42, 4.02]). Stakeholder involvement also scored significantly higher than Rigour of development (*MD* = 1.25, *SE* = 0.05, *p* < .001, 95% *CI* [1.10, 1.39]) and Editorial independence (*MD* = 1.76, *SE* = 0.11, *p* < .001, 95% *CI* [1.42, 2.09]), but significantly lower than Clarity of presentation (*MD* = − 1.77, *SE* = 0.07, *p* < .001, 95% *CI* [–1.97, − 1.57]). However, there was no statistically significant difference between Stakeholder involvement and Applicability (*MD* = 0.02, *SE* = 0.04, *p* = 1.00, 95% *CI* [–0.11, 0.15]).

## Discussion

The analysis revealed statistically significant differences across the six aspects of the World Health Organisation Disease Outbreak News guidelines evaluated in this study. While the guidelines scored highly in Scope and Purpose and Clarity of Presentation, they performed markedly lower in Editorial Independence and Rigour of Development, indicating imbalances in methodological rigour and transparency. These findings have implications for the perceived trustworthiness, transparency, and practical utility of the guidelines. During disease outbreaks, when effective containment and management depend heavily on clear and credible guidelines, even isolated weaknesses, such as insufficient rigour in development or limited stakeholder involvement, can significantly undermine confidence in and the practical applicability of the guidance provided. The large effect size observed in this study (*ηp²* = 0.866) signals that the disparities across domains are not trivial but carry meaningful implications for public health governance.

The consistently high scores for Scope and Purpose (*M* = 6.46, *SD* = 0.45) and Clarity of Presentation (*M* = 6.27, *SD* = 0.68) reflect WHO’s strength in articulating clear objectives and producing user-friendly guidelines. These results echo earlier studies that underline the importance of comprehensibility and goal clarity in public health communication [1, 13]. In times of health crisis, well-scoped and clearly articulated guidelines are essential for mobilising a coordinated response and ensuring rapid uptake among public health authorities, clinicians, and the general public [7]. The commitment of the WHO to improving clarity and defining the intent of each guideline, enshrined in its Handbook for Guideline Development [12], appears to be well implemented in this area.

However, the domain Editorial Independence received the lowest score (*M* = 2.74, *SD* = 1.02), with statistically significant differences observed when compared to all other domains (e.g., Scope and Purpose, *MD* = 3.72, *p* < .001). This finding shows long-standing concerns about transparency in the development process of WHO guidelines. As argued by Taylor et al. [15], the processes involved in the formulation of WHO guidelines “often lacked transparency”. Given that DONs often serve as the primary source of information for governments, media, and the public, any perception of undue political, institutional, or donor influence can significantly undermine trust in the guidance provided. In high-stakes scenarios where rapid decision-making is required, the credibility of the guidelines depends not only on the quality of the evidence but also on the assurance that the recommendations are free from bias and external interference. The low score in this domain suggests that despite the establishment of the WHO Guideline Review Committee (GRC) to strengthen oversight and credibility, the organisation may need to intensify its efforts to reassure stakeholders of its editorial independence, especially during politically sensitive global health emergencies and to ensure transparency in the mechanisms that underline the formulation of its guidelines.

The Rigour of Development domain recorded a relatively low mean score (*M* = 3.26, *SD* = 0.75), with statistically significant differences observed when compared to higher-performing domains such as Scope and Purpose (*MD* = 3.21, *p* < .001) and Clarity of Presentation (*MD* = − 3.02, *p* < .001). This finding raises critical concerns about the scientific robustness of guidelines developed under emergency conditions. The results align with those reported by Taylor et al. [15], who observed that WHO guidelines often rely heavily on qualitative evidence, with limited transparency regarding the synthesis and integration of that evidence during development. Although the need for rapid guideline development during fast-evolving outbreaks is understandable, the trade-off between speed and methodological rigour warrants careful consideration. As Garritty et al. [6] argue, evidence-based guidelines should be grounded in systematic, transparent, and reproducible methodologies to ensure reliability. The deficiencies observed in this domain may partly reflect the practical constraints inherent in emergency response scenarios; however, they also highlight the need for structural reforms, such as the pre-establishment of rapid review protocols that preserve methodological integrity even under time pressure.

It is worth noting, however, that the low performance in this domain may also be partly contextual. Many of the documents disseminated through the Disease Outbreak News (DONs) platform are not formal guidelines in the strict methodological sense but rather rapid guidance documents issued under the urgent conditions of public health emergencies. These guidance documents are often developed to provide interim recommendations in the absence of complete or fully appraised evidence and are intended to support swift decision-making by national and local health authorities. Additionally, many of the WHO emergency guidelines examined in this study are embedded within Disease Outbreak News (DONs) bulletins, which primarily serve as real-time communication tools. These bulletins provide a wealth of epidemiological and situational data yet often omit explicit references to the underlying evidence base from which specific guideline recommendations are drawn. As such, the apparent weakness in methodological rigour may not necessarily reflect the absence of a sound evidence base, but rather the limited visibility of these processes within the final published product. This highlights an important distinction between the operational purpose of the DONs and the evaluative criteria typically used to assess guideline quality and suggests a need for greater transparency in linking recommendations to supporting evidence, even in emergency communications.

The moderate scores for Stakeholder Involvement (*M* = 4.50, *SD* = 0.69) and Applicability (*M* = 4.48, *SD* = 0.70) also warrant attention. These domains were not significantly different from each other (*p* = 1.00), indicating similar levels of performance. Yet both scored significantly lower than Scope and Purpose, suggesting that although the WHO excels in defining the scope of guidelines, it may fall short in involving end-users and ensuring the practical application of recommendations. This aligns with literature noting the challenges of broad stakeholder engagement in emergency contexts [13, 14]. Inclusive guideline development that incorporates local practitioners, affected communities, and regional actors enhances contextual relevance and promotes adherence. The participation of stakeholders can improve not only the implementation feasibility or applicability of DONs recommendations but also strengthen public trust, promote adherence, and reduce resistance to public health interventions. In this regard, stakeholder engagement is not simply a procedural requirement but a strategic necessity for effective emergency response. A more participatory approach that systematically integrates feedback from local actors and vulnerable populations should therefore be institutionalised within the drafting process of disease outbreak guidelines to ensure that the guidance produced is both scientifically sound and socially grounded, particularly in settings where time-sensitive, context-specific responses are critical.

From a broader perspective, these findings reinforce the essential role of high-quality guidelines in effective outbreak response. As established in the literature, disease outbreaks create an urgent demand for decision-making amid uncertainty and limited evidence [5, 8]. WHO guidelines are intended to provide structure and legitimacy to these decisions, as stipulated under the International Health Regulations (2005) and operationalised through tools such as Disease Outbreak News (DONs). However, as seen in previous crises, including Ebola and COVID-19, lapses in communication, stakeholder engagement, and editorial transparency can undermine global efforts to contain outbreaks [9, 10].

## Conclusion

Given the critical role WHO guidelines play in shaping global responses to health emergencies, enhancing the quality of these documents is not only necessary but urgent. The effectiveness of public health interventions during outbreaks depends heavily on the credibility, clarity, and practical utility of such guidelines. Addressing the weaknesses identified in this study, especially in the domains of editorial independence and stakeholder involvement, will improve trust, foster better compliance, and ultimately contribute to more effective global health responses. As public health threats continue to evolve, the WHO must ensure that its guideline development processes are not only fast and responsive but also balanced, inclusive, and methodologically sound. Future efforts should aim to institutionalise these improvements to better prepare for and respond to emerging health risks.

## Data Availability

No datasets were generated or analysed during the current study.
